# Mode of action of fluopyram in plant-parasitic nematodes

**DOI:** 10.1038/s41598-022-15782-7

**Published:** 2022-07-13

**Authors:** A. Sylvia S. Schleker, Marc Rist, Christiane Matera, Arunas Damijonaitis, Ursel Collienne, Koichi Matsuoka, Samer S. Habash, Katja Twelker, Oliver Gutbrod, Corinna Saalwächter, Maren Windau, Svend Matthiesen, Tatyana Stefanovska, Melanie Scharwey, Michael T. Marx, Sven Geibel, Florian M. W. Grundler

**Affiliations:** 1 Molecular Phytomedicine, University of Bonn, Karlrobert-Kreiten-Straße 13, 53115 Bonn, Germany; 2 Research and Development, CropScience Division, Bayer AG, Alfred-Nobel-Str.50, 40789 Monheim am Rhein, Germany; 3 Department of Entomology, National University of Life and Environmental Sciences, Kyiv, 03041 Ukraine; 4 Present Address: BASF Vegetable Seeds, Napoleonsweg 152, 6083 AB Nunhem, The Netherlands

**Keywords:** Molecular biology, Parasitology

## Abstract

Plant-parasitic nematodes (PPN) are responsible for severe yield losses in crop production. Management is challenging as effective and safe means are rare. Recently, it has been discovered that the succinate dehydrogenase (SDH) inhibitor fluopyram is highly effective against PPN while accompanying an excellent safety profile. Here we show that fluopyram is a potent inhibitor of SDH in nematodes but not in mammals, insects and earthworm, explaining the selectivity on molecular level. As a consequence of SDH inhibition, fluopyram impairs ATP generation and causes paralysis in PPN and *Caenorhabditis elegans*. Interestingly, efficacy differences of fluopyram amongst PPN species can be observed. Permanent exposure to micromolar to nanomolar amounts of fluopyram prevents *Meloidogyne* spp. and *Heterodera schachtii* infection and their development at the root. Preincubation of *Meloidogyne* *incognita* J2 with fluopyram followed by a recovery period effectively reduces gall formation. However, the same procedure does not inhibit *H.* *schachtii* infection and development. Sequence comparison of sites relevant for ligand binding identified amino acid differences in SDHC which likely mediate selectivity, coincidently revealing a unique amino acid difference within SDHC conserved among *Heterodera* spp. Docking and *C.* *elegans* mutant studies suggest that this minute difference mediates altered sensitivity of *H.* *schachtii* towards fluopyram.

## Introduction

Plant-parasitic nematodes (PPN) cause substantial damage in a broad spectrum of economically relevant crops. Global annual yield losses are estimated to be 12.3% on average or more than 100 billion US dollar^1–4^. Sedentary PPN comprising cyst (*Heterodera* spp., *Globodera* spp.) and root-knot nematodes (*Meloidogyne* spp.) are of highest significance in this respect. Thus, nematode control is necessary to ensure sustainable crop production, though challenging as non-selective nematicides or fumigants are banned in many countries due to their unfavorable safety profiles. In the last years, significant progress has been made towards discovering and introducing new nematicides with novel modes-of-action, not only reducing dose rates per hectare but also promising to be more selective and thus potentially less harmful for non-target organisms and the environment. Of these, fluopyram was initially registered as a fungicide^5^ and assessed to be of low and hence acceptable regulatory risk for mammals, bees, the examined arthropods, earthworms, plants, soil and aquatic organisms^6^.

Fluopyram is reported to impact PPN: In vitro fluopyram reduces hatching of *Meloidogyne incognita*, *Heterodera glycines*, and *Heterodera schachtii* but not of *Caenorhabditis elegans* as the egg shell possesses limited permeability for the compound^7,8^. Fluopyram is reported to be of acute toxicity for *M.* *incognita*, *M. javanica* and *H.* *schachtii* infective juveniles (J2)^8,9^. *Meloidogyne* *incognita* and *Rotylenchulus reniformis* recover from fluopyram treatment whereas *C.* *elegans* does not^7,10^. Studies of Wram and Zasada^11^ show acute toxicity of fluopyram against *M.* *incognita* but no effects after short term exposure to sublethal concentrations^11^. However, general methods, compound dose and exposure time varies between the different reports and experiments. Thus, comparability of the results is limited and individual conclusions need to be seen as specific for the applied conditions.

Fluopyram was discovered to inhibit the mitochondrial complex II of the aerobic respiratory chain in fungi and *C.* *elegans*^7,12,13^. The target protein is a transmembrane complex comprised of four protein subunits (SDHA to D), a flavine adenine dinucleotide (FAD) cofactor (bound to SDHA), three iron-sulfur clusters (within SDHB) and a heme moiety (between SDHC and D). The succinate binding site is within SDHA. The ubiquinone (coenzyme Q) binding pocket is blocked by fluopyramat the interface of SDHB, C and D (Fig. S1). Complex II possesses both succininate:ubiquinone oxidoreductase (SQR) and succiniate dehydrogenase (SDH) activity. In literature, fluopyram is referred to as a succinate dehydrogenase inhibitor (SDHI)^12^. Thus, for simplicity the acronym SDH will be used to refer to the mitochondrial complex II.

Here we investigated the impact of fluopyram on the root-knot nematodes *M.* *incognita* and *M.* *javanica* as well as the cyst nematode *H.* *schachtii* in detail. Extreme differences in sensitivity of the two genera towards the compound were observed. In our study we aim at linking target features to the monitored differences in vivo. While fluopyram inhibition data on the molecular target, the complex II of the mitochondrial electron transport chain in fungi and *C.* *elegans* have been published^5,12^, detailed studies on the molecular (or biochemical) mode-of-action in PPN and in non-target organisms are missing, limiting a detailed understanding of the molecular determinants of selectivity.

## Results

### *Heterodera schachtii* is far less sensitive to transient fluopyram exposure than *Meloidogyne* spp

**Figure 1:**
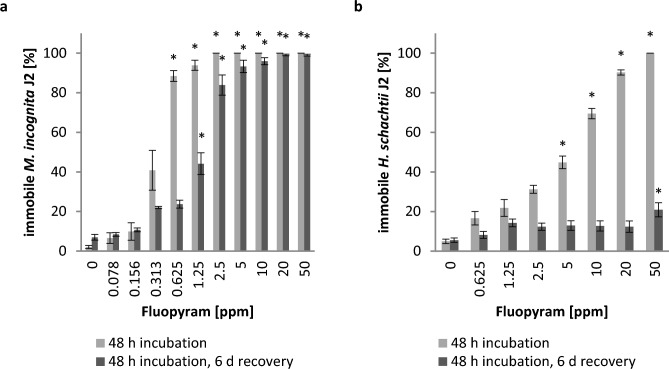
Fluopyram is lethal for *Meloidogyne incognita* but not for *Heterodera schachtii*. *M.* *incognita* J2 (**a**) and *H.* *schachtii* J2 (**b**) were exposed to different concentrations of fluopyram or DMSO control for 48 h. Subsequently, nematodes were washed and incubated in water for further 6 days. The number of immobile nematodes (without (**a**) or with NaOH stimulus (**b**)) were counted at both of these time points. Values represented as mean ± SE of three independent biological replicates (n = 6–12 (**a**); n = 14–18 (**b**)). Asterisks indicate significant differences to control according to Dunn’s method (**b**) (*p* < 0.05).

We first investigated the direct effect of fluopyram on the root-knot nematode *M.* *incognita* and the cyst nematode *H.* *schachtii*. Exposing the second stage infective juveniles (J2) of *M.* *incognita* to different concentrations of fluopyram for 48 h revealed that 0.36 ppm (0.9 µM) fluopyram was sufficient to paralyze 50% of the nematodes. To obtain the same effect on *H.* *schachtii* J2, an about 14 times higher concentration of fluopyram (4.9 ppm/12.4 µM) was necessary. To determine whether the fluopyram-induced paralysis is reversible, we subsequently removed the compound and incubated the J2 in water for 6 days. 50% of the *M.* *incognita* J2 recovered from a fluopyram pre-treatment with 1.5 ppm (3.8 µM), while more than 90% were irreversibly affected by 5 ppm (12.6 µM) fluopyram. Interestingly, *H.* *schachtii* J2 fully recovered from fluopyram pre-treatments up to 20 ppm (50 µM). Despite 100% paralysis at 50 ppm (126 µM), only 21% remained immotile after the recovery phase (Fig. 1).

**Figure 2:**
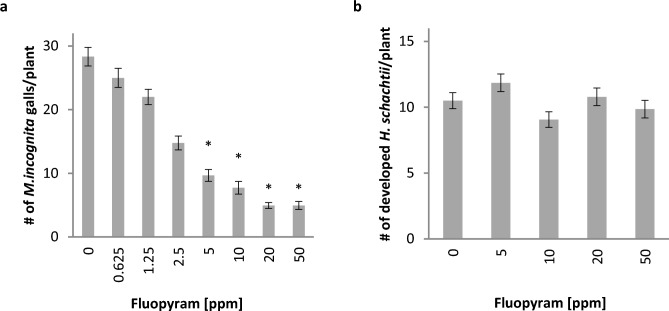
Pre-treatment of J2 with fluopyram reduces *Meloidogyne incognita* caused gall formation but not *Heterodera schachtii* infection of host plants. *M.* *incognita* J2 (**a**) and *H.* *schachtii* J2 (**b**) were incubated in different concentrations of fluopyram or DMSO as control for 48 h. Subsequently, the compound and DMSO were removed, the J2 were incubated in water for further 6 days and then used to inoculate lettuce (*M.* *incognita*) or *Arabidopsis thaliana* (*H.* *schachtii*). The number of galls (*M.* *incognita*) or the number of males and females (*H.* *schachtii*) was determined. Bars display mean ± SE of three independent biological replicates (n = 17–18 (**a**); n = 56–60 (**b**)). Asterisks indicate significant differences to control according to Dunn’s method (*p* < 0.05).

To assess the virulence of the fluopyram-treated J2 and validate the obtained recovery data, we inoculated lettuce or *A. thaliana* with the 48 h incubated and 6 days recovered *M.* *incognita* or *H.* *schachtii* J2 (Fig. 2). As expected, this treatment efficiently reduced the number of galls caused by *M. incognita* infection. Comparable effects were observed for *M. javanica* (Fig. S2). In contrast, transient exposure to fluopyram did not impact *H. schachtii* J2 infectivity and development of adult nematodes. Whereas 2.5 ppm (7.3 µM) fluopyram reduced the number of galls by 48%, even 50 ppm (126 µM) fluopyram did not alter the number of mature *H.* *schachtii* males and females and thus infection events compared to the control (Fig. 2).

### Continuous exposure to nanomolar concentrations of fluopyram prevents *H. schachtii* infection and development

**Figure 3:**
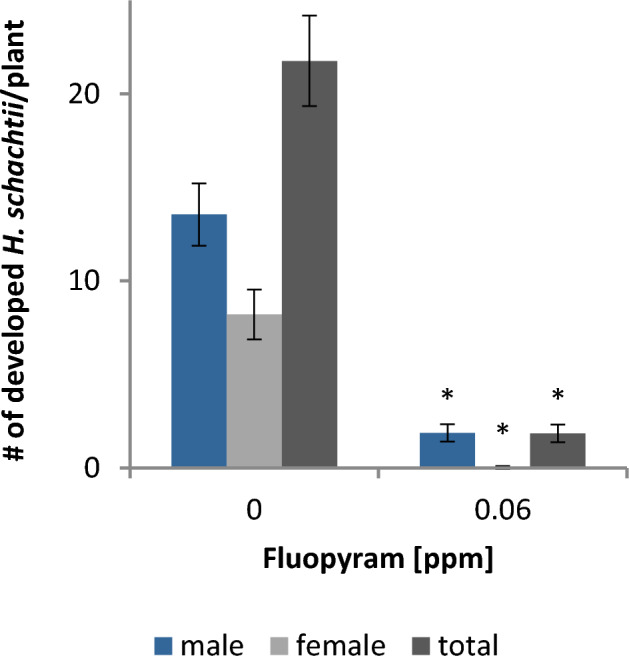
Fluopyram in the plant growth medium efficiently protects plants from *Heterodera schachtii*. *Arabidopsis thaliana* was grown on fluopyram- or DMSO-containing agar medium. Plants were inoculated with *H.* *schachtii* J2 and the number of adult males and females was determined. Bars display mean ± SE of three independent biological replicates (n = 33–38). Asterisks indicate significant differences to DMSO control according to Dunn’s method (*p* < 0.05).

Although fluopyram is not nematicidal for *H.* *schachtii* J2 after transient exposure to tested rates, the compound paralyzes J2. Therefore, we investigated if permanent exposure of the nematodes to fluopyram in the plant growth medium has an impact on *H.* *schachtii* parasitism of *A. thaliana*. Under these conditions fluopyram inhibited J2 motility in a concentration dependent manner. When the plant growth medium was supplemented with 5 ppm (12.6 µM) fluopyram most J2 did not move out or far from the point of inoculation. In the presence of 1.25 ppm (3.2 µM) fluopyram J2 were able to move towards the root but did not invade the root tissue. We observed very few infection events and no further development when J2 were permanently exposed to 0.313 ppm (0.78 µM) fluopyram. At a concentration of 0.06 ppm (0.15 µM) fluopyram J2 were able to invade the root tissue but only very few were capable to develop into males or females. Under these conditions, the average number of developed males per plant was reduced by 91.5% and the average number of females per plant was reduced by 99.6% compared to the control (Fig. 3).

### Fluopyram interferes with ATP biosynthesis in nematodes

**Figure 4:**
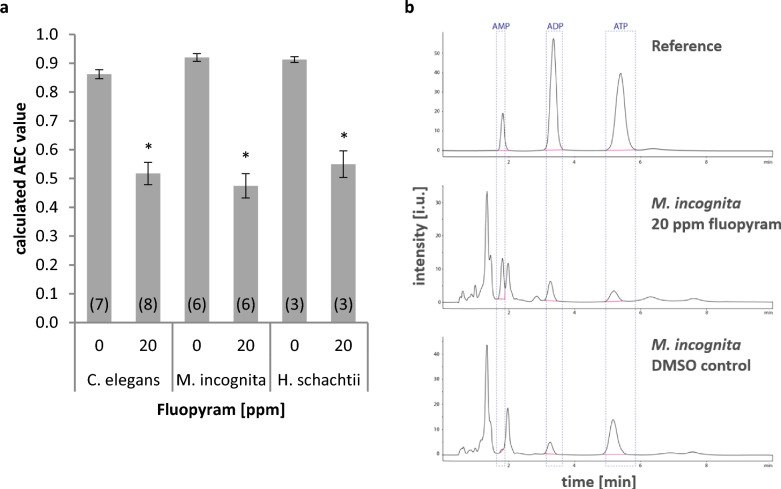
Impact of fluopyram on ATP generation of *Caenorhabditis elegans*, *Meloidogyne incognita* and *Heterodera schachtii*. Nematodes were incubated in DMSO or 20 ppm fluopyram for 48 h prior to measurement of adenylate energy charge (AEC). (**a**) AEC values were calculated from HPLC-traces. Values represented as mean ± SE of duplicates from three to eight independent biological replicates (indicated in brackets at the bottom of each bar). Asterisks indicate significant difference between DMSO control and treatment of each nematode species according to Holm-Sidak method (*p* < 0.05). There is no significant difference between the treated variants. (**b**) Representative HPLC-traces. *p* < 0.05.

To link target and in vivo data and to better understand the observed difference in sensitivity and phenotype (paralysis/relaxation) of *M.* *incognita* and *H.* *schachtii* towards fluopyram, we performed an analysis of the energy state. Measuring relative amounts of ATP, ADP and AMP in organisms reveals potential effects of a molecule on energy metabolism. In order to validate that ATP biosynthesis is impaired by fluopyram in the different nematode species, we chose 20 ppm (50 µM) fluopyram for this experiment as a 48 h incubation resulted in immobilization rates of 90% (*H.* *schachtii*) and 100% (*M.* *incognita*, *C.* *elegans*) (Fig. 1, 7a). Incubation with fluopyram (20 ppm/50 µM for 48 h) resulted in reduced ATP peak areas and thus low adenylate energy charge (AEC) values in all three nematode species (Fig. 4). Compared to the untreated control fluopyram treatment caused a 40% reduction of the AEC value in *C.* *elegans* and *H.* *schachtii*, and a reduction by 49% in *M.* *incognita*. The absolute AEC values were 0.52, 0.55, and 0.47 for *C.* *elegans*, *H.* *schachtii*, and *M.* *incognita*, respectively. AEC values were not significantly different between the three species at the used compound concentration and exposure time but we observed a tendency for a greater reduction in *M.* *incognita*. These data clearly indicate that fluopyram interferes with ATP biosynthesis in the tested nematode species. .

### Fluopyram is a highly selective inhibitor of the *C. elegans* SDH

**Figure 5:**
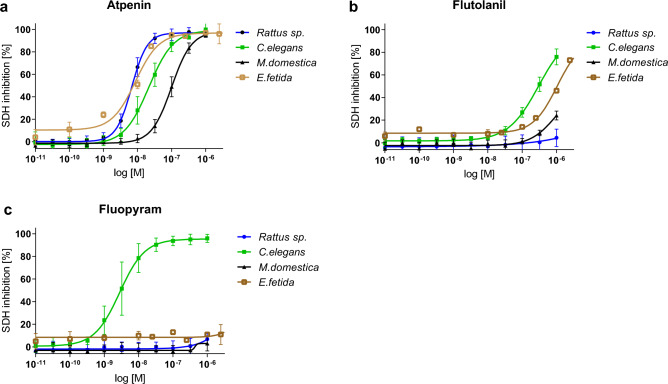
Inhibition of SDH activity. The efficacy of atpenin (**a**), flutolanil (**b**) and fluopyram (**c**) in inhibiting SDH activity was measured using mitochondrial preparations from house fly (*Musca domestica*, black line), rat (*Rattus* sp., blue line), earthworm (*Eisenia fetida* subsp. *andrei*, brown line), and nematode (*C. elegans*, green line). Values represented as mean ± SE of duplicates from three or four independent biological replicates. The following pIC_50_ values were calculated: (**a**) SDH of *C. elegans*, rat, fly, and earthworm were inhibited with an efficacy of pIC_50_ = 7.7, 8.2, 7.0 and 8.1, respectively, by Atpenin A5. **(b)** Flutolanil possesses a pIC_50_ of 6.5 and 6.0 against *C. elegans* and *E. fetida*, respectively. (**c**) Fluopyram inhibits nematode SDH with an efficacy of pIC_50_ = 8.5. pIC_50_ values are not given in case they are outside the measured concentration range.

To better understand the molecular determinants of activity differences we assessed the compound’s effects on the putative molecular target SDH in more detail. Three known SDH inhibitors were tested to determine species-selectivity and respective efficacy using mitochondrial preparations from *Musca domestica* (house fly), the free-living nematode *C. elegans*, the earthworm species *Eisenia fetida* subsp. *andrei*, and *Rattus* sp. (rat) (Fig. 5).

As expected, the non-selective complex II inhibitor atpenin A5 showed strong SDH inhibition independent of the species. In contrast, the fungicide flutolanil was not active against the mammal SDH and only active against the SDH of *C. elegans*, *E. fetida* and house fly at highest concentrations. Interestingly, fluopyram was extremely potent for the *C. elegans* SDH (pIC_50_ = 8.5) and two orders of magnitude more selective for *C. elegans* SDH than flutolanil. In addition, fluopyram did not inhibit the complex II of other test species within the applied concentration range.

### SDHC amino acid substitution mediates nematode insensitivity towards fluopyram

**Figure 6:**
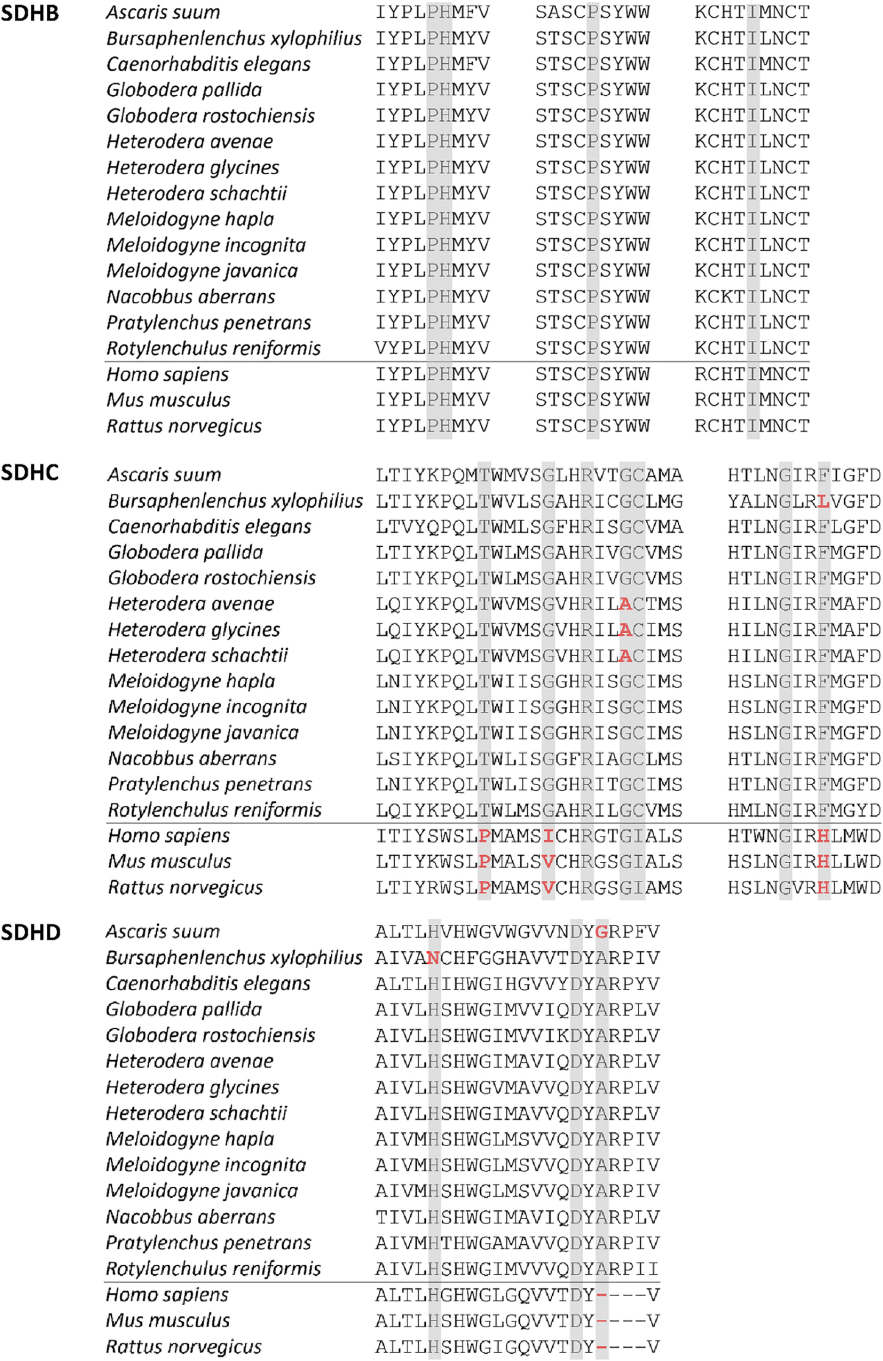
SDHB, SDHC and SDHD sequence comparison. Sequence parts and residues (highlighted in grey) identified to be involved in ligand binding in *Caenorhabditis elegans* according to Burns et al.^13^ are displayed^13^. Differences to *C. elegans* are highlighted in red.

A *C.* *elegans* resistance screen highlighted that mutations of certain amino acids of the SDHB, SDHC and SDHD protein subunits of complex II mediate nematode insensitivity to wact-11^13^. The wact-11 family core structure and, in particular, wact-11 is closely related to that of fluopyram. Our sequence comparison of the relevant SDHB, SDHC and SDHD regions elucidated an amino acid exchange from G to A at position 90 in the *H.* *schachtii* SDHC subunit. Further analyses revealed that this amino acid difference is conserved within *Heterodera* spp. but could not be observed in any other nematode species analyzed, including *Globodera* spp. Besides *Heterodera* spp., *B.* *xylophilus* is the only other PPN we analyzed that harbors amino acid substitutions in two of the as relevant described sites (Fig. 6).

**Figure 7:**
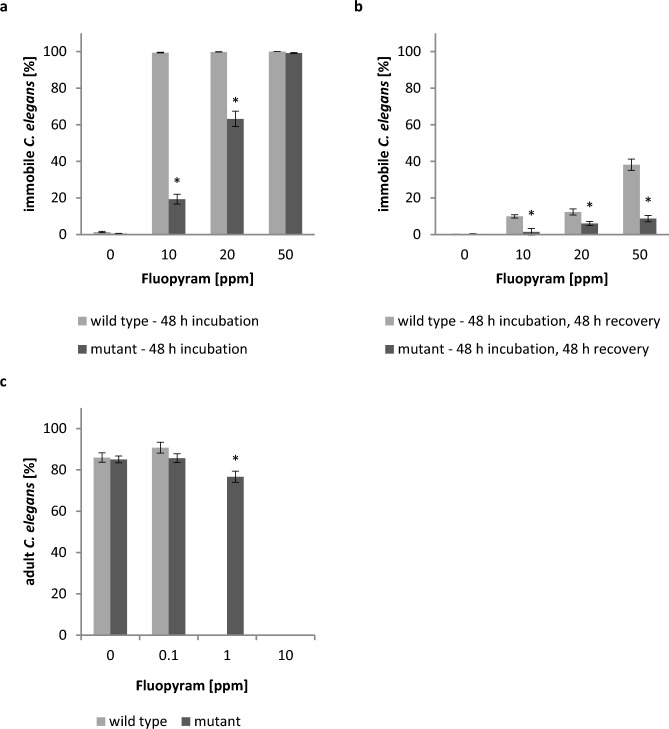
SDHC G77D mutation reduces *Caenorhabditis elegans* sensitivity against fluopyram. Synchronized *C.* *elegans* L1 were exposed to different concentrations of fluopyram or DMSO control for 48 h. Subsequently, nematodes were washed and incubated in water for 48 h. The number of immobile nematodes was counted at both of these time points (**a**,**b**). The number of nematodes that developed from L1 into adults was determined after permanent exposure to fluopyram or DMSO control for 6 days (**c**). Bars display mean ± SE of at least six (**a**,**b**) or three (**c**) independent biological replicates (n = 23–36 (**a**,**b**); n = 11–12 (**c**)). Asterisks indicate significant differences between wild type and mutant at each condition according to Dunn’s method (*p* < 0.05).

To validate that this amino acid difference observed in *H.* *schachtii* SDHC mediates increased tolerance towards fluopyram, we tested the susceptibility of a suitable corresponding *C.* *elegans* mutant (strain RP2699 with the G77D residue change in SDHC) to fluopyram. The mutant was significantly less sensitive to fluopyram compared to the *C.* *elegans* wild type. When incubated with 10 ppm (25 µM) fluopyram for 48 h more than 99% of the wild type nematodes were inactive while only about 19% of the juveniles harboring the mutation were affected (Fig. 7a). Although more than 99% of the *C.* *elegans* mutant nematodes were inactive after 48 h in 50 ppm (126 µM), they nearly completely recovered with less than 9% remaining immobile. In contrast, 100% of the *C.* *elegans* wild type nematodes were immobile after 48 h incubation in 50 ppm with this effect being nonreversible for about 38% (Fig. 7b). Permanent exposure of *C.* *elegans* L1 stage juveniles to 1 ppm (2.5 µM) fluopyram completely inhibited maturation of the wild type nematodes but not of the mutant strain. Compared to the untreated mutant strain control the percentage of mutant juveniles exposed to 1 ppm fluopyram reaching adulthood after 6 days was reduced by 10% only with no statistically significant difference compared to the control (Fig. 7c).

### Protein homology modeling of nematode SDH sequences

**Figure 8:**
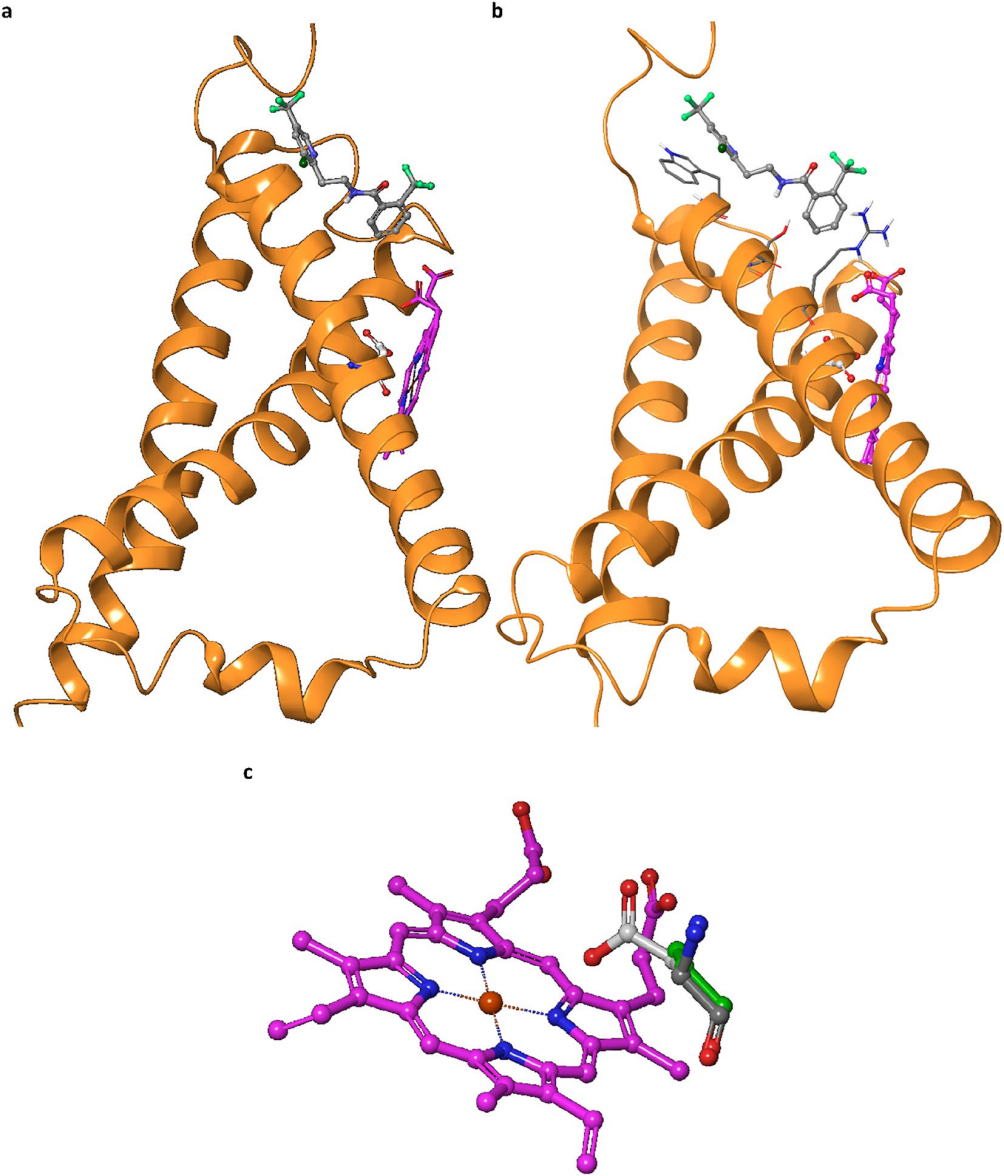
Amino acid difference in SDHC could cause conformational change. (**a**) SDHC of *Caenorhabditis elegans* SDH model with heme moiety and manually docked fluopyram. The resistance position (Asp instead of Gly in the utilized *C. elegans* mutant) is in close proximity (< 3.0 Å) to the porphyrin ring and about 9 Å apart from the assumed fluopyram binding position. (**b**) Alignment of pivotal residues on the edge of first transmembrane helix in SDHC with respect to the heme moiety and the assumed fluopyram binding model; displayed are residues Trp-67, Ser-70, Arg-74 and the resistance mutation position Asp-77 (from left to right), which are almost perfectly aligned on the edge of SDHC’s first transmembrane helix. (**c**) Close-up of the resistance mutation site (Asp in white, Ala in green, Gly in dark grey) shows that the resistance mutation side chain—in particular Asp—modulates both sterically and electronically the porphyrin moiety (shortest distance < 3.0 Å) which may lead to some conformational reorientation of the catalytic site.

Homology modeling studies based on the nematode SDHB, C and D sequences suggested the resistance mutation site to be placed about 9 Å apart from the assumed fluopyram binding site, in close proximity to one of the porphyrin rings of the catalytic heme. It is located in the middle of the first transmembrane helix of SDHC, with the sidechain pointing to the heme scaffold (Fig. 8a).

This SDHC helix plays a pivotal role in both substrate and inhibitor binding as well as aligning the heme moiety properly: Trp-sidechain is part of the hydrophobic fluopyram binding site, Ser-OH is involved in H-bonding, and Arg- is required for the correct heme alignment by forming a salt bridge with the carboxylic groups of the porphyrin rings (Fig. 8b).

A direct interaction between the fluopyram binding and the mapped resistance site G77D is unlikely owing to the relatively long distance of about 9 Å between the two regions. As pointed out, the putatively resistance-conferring position, in particular its sidechain is in close contact with one of the porphyrin bridges. Compared to the sterically undemanding Glycine introduction of an Alanine (introduction of an additional methyl group) and moreover Aspartate (insertion of a propionic acid with a negative charge) may alter the arrangement of the heme anchoring (Fig. 8b,c). A subsequent realignment of this pivotal helix may then affect fluopyram binding or access to the binding site.

## Discussion

Fluopyram has recently been discovered to control PPN and commercial products for agricultural uses are available. Although there are several studies showing activity of fluopyram against nematodes, detailed mode-of-action studies in PPN are missing. Here we show that fluopyram targets the complex II of the electron transport chain in nematodes and inhibits ATP generation. Fluopyram selectively inhibits SDH function in nematodes but not in non-target organisms such as rat, fly, and the earthworm species *Eisenia fetida* subsp. *andrei*. Exposure of nematodes to fluopyram causes reversible or irreversible paralysis in a concentration dependent manner revealing remarkable differences in sensitivity of *Meloidogyne* spp. and *H.* *schachtii* towards the compound. We identified a single amino acid difference within SDHC between *Heterodera* spp. and *Meloidogyne* spp. at a position crucial for interaction of fluopyram with its target. This genetic difference likely renders *H.* *schachtii* to be less sensitive towards the compound as validated by *C.* *elegans* mutant studies.

Exposure of nematodes to a test substance often causes inactivity of the organism and even inability to respond to external stimuli although the nematodes are not dead. To overcome this limitation and avoid misinterpretation of observations we incubated the J2 in compound-free medium or water (recovery phase) after a 2-day fluopyram treatment. Additionally, we investigated to what extent the infectivity of the treated PPN after the recovery phase was impaired. This experimental setup provides highly reliable data for making a statement on the antihelmintic properties of a compound. Secondly, it makes laborious microscopic phenotyping of compound-induced damages unnecessary. Utilizing this procedure, we observed great differences in the sensitivity of *M.* *incognita*, *M.* *javanica* and *H.* *schachtii* J2 towards fluopyram. Fluopyram is nematicidal for *Meloidogyne* spp*.* For *M.* *incognita* J2 the EC_50_ (48 h treatment, 6 days recovery) was as low as 1.5 ppm. Further, J2 pre-treatment with 2.5 ppm fluopyram was sufficient to reduce gall formation by 48% and 20 ppm fluopyram resulted in more than 80% gall reduction. This shows that *M.* *incognita* J2 are irreversibly inhibited by fluopyram in a concentration dependent manner and thus fluopyram is nematicidal for *M.* *incognita* at low compound concentrations. These results are in line with Faske and Hurd^10^ who showed that *M.* *incognita* J2 are very sensitive to fluopyram treatment as 50% of the J2 are immobile after incubation in 1.18 ppm for 24 h^10^. Despite strong activity after permanent exposure, we found that fluopyram is nematistatic for *H. schachtii* at concentrations up to 20 ppm as treated J2 were able to recover. Although nematicidal activity of the compound is statistically significant at 50 ppm, we only documented a 21% paralysis rate of 48 h treated (50 ppm fluopyram) and 6 days recovered *H.* *schachtii* J2 in vitro. There was no impact on the number of developed nematodes on *A.* *thaliana* roots after transient exposure to fluopyram for 48 h. Kim et al.^8^ report that fluopyram is lethal for *H.* *schachtii* J2 with a 24 h LC_50_ of 0.0543 ppm^8^. Reasons that possibly explain the differences between the reported LC_50_ values and our observations and the conclusions are as follows: First, the authors do not specify how they decided whether a J2 is dead or not. We found that sodium hydroxide is a potent stimulating agent to trigger J2 movement which is especially helpful when evaluating the effect of a compound on *H.* *schachtii* J2 in in vitro assays. Secondly, the authors did not investigate whether the treated J2 are able to recover and able to infect a host plant. In general, it would be desirable to have a coordinated protocol to precisely determine the nematicidal or nematistatic activity of a compound in order to ascertain comparability of data generated in different institutions. To what extend nematistatic or nematicidal properties of a compound will affect its potential for nematode management in practice remains to be tested but will likely depend on its concentration and half-life in the soil.

In order to approach a mechanistic explanation for the observed differences between the nematode species we first determined the impact of fluopyram on ATP biosynthesis in *C.* *elegans*, *M.* *incognita* and *H.* *schachtii*. As ATP is essential for cellular processes including muscle contraction, fluopyram-induced ATP loss causes paralysis of nematodes. Prolonged ATP depletion to low levels leads to irreversible cell damages and finally to death of the organism. The AEC value is considered as a parameter that categorizes an organism to have normal metabolic activity at a value between 0.7 and 0.95. AEC values below 0.5 are associated with irreversible damage and thus cell death. At AEC values between 0.5 and 0.8 the cell/organism may recover from the stress situation as it is considered to stay viable^14^. We proved that fluopyram interferes with ATP biosynthesis in all three nematodes to a similar extent which is in accordance with our paralysis data. Although the AEC values between fluopyram-treated nematode species did not significantly differ, the AEC values for *C.* *elegans* and *H.* *schachtii* were above 0.5 whereas it dropped below this threshold for *M.* *incognita*. This could explain our observation that only *M.* *incognita* J2 were not able to recover from the transient fluopyram treatment. As inhibition of respiratory chain/mitochondrial electron transport chain activity leads to reduced levels of ATP we were interested if fluopyram alters the activity of a molecular target in the electron transport chain and whether there are differences between nematodes and non-target organisms. Not having a functional assay to test PPN species we analyzed *C.* *elegans* as well as rat, fly and the earthworm *E. fetida*. We demonstrate that micromolar to nanomolar concentrations of fluopyram selectively inhibit complex II function of *C. elegans* which is not the case for rat, house fly and *E. fetida*.

This is in accordance with Burns et al.^13^ who reported that fluopyram impairs complex II activity of the *C.* *elegans* wild type (N2)^13^. Additionally, the structurally related wact-11 compound was shown to be active against *C.* *elegans* complex II but not against mouse complex II^13^. Moreover, our comparison of selected amino acid sequences of complex II *sdh* genes revealed that human, mouse and rat are very similar on genetic level, especially revealing amino acid differences compared to nematodes at three positions within SDHC known to be important for interaction of fluopyram with the target (Fig. 6, highlighted in red). Based on these data we are able to understand the molecular determinants of selectivity and provide target-based evidence that fluopyram does not possess activity against the SDH of mammals, insects and earthworm but is highly selective for nematodes and fungal pathogens.

It is known that several SDH residues are important for binding of SDHIs to its target and are conserved among nematodes^13^. By aligning the respective amino acid sequence regions among several PPN we identified a single amino acid difference within *Heterodera* spp. at a crucial SDHC residue. We further showed that a *C.* *elegans* mutant strain with a point mutation at this position is much less sensitive towards fluopyram treatment compared to the wild type. After 48 h incubation in 10 ppm fluopyram nearly 100% of the wild type nematodes were immobile. In contrast, about 5 times less mutant nematodes were affected. Transient incubation in 50 ppm fluopyram immobilized all/nearly all wild type and mutant nematodes. However, approx. 90% of the mutant nematodes were able to recover—4.4 times more than for the wild type. Thus, our data indicate that this genetic difference between *Heterodera* spp. and *Meloidogyne* spp. is at least one major reason for the observed differences in sensitivity towards fluopyram. Further evidence is given by our in silico modeling studies showing that the amino acid difference is not located directly at the fluopyram binding pocket but could lead to steric reorganization of SDHC and thus indirectly interfere with ligand binding. Besides this, morphological characteristics like cuticle thickness, permeability and turnover rate of cyst and root-knot nematode J2 may play a role as well as discussed earlier to explain differences in sensitivity of these PPN towards fluazaindolizine^15^. Interestingly, we observed that *C.* *elegans* unlike *M.* *incognita* is able to recover from fluopyram treatment indicating that other so far not identified amino acids in the target might be important for ligand interaction and proper target function. In addition, other factors such as metabolic detoxification mechanisms and/or morphological differences could be involved as well. Another PPN genus that would be interesting to investigate is *Bursaphelenchus* spp. as we identified two amino acid differences within SDHC of *B.* *xylophilius* at positions important for ligand interaction. The fact that single amino acid differences can confer complete insensitivity of an organism towards a substance is established knowledge and thus supports our findings. In particular—utilizing a *C.* *elegans* mutant screening approach—it was demonstrated that amino acid differences within complex II proteins of *C.* *elegans* are responsible for variations in sensitivity of the nematode towards respective inhibitors^13^. As fluopyram was initially introduced as a fungicide in 2009, additional evidence is provided by studies reporting the emergence of resistant fungal strains of usually sensitive species due to individual amino acid substitutions^16^ and investigations attesting that certain mutations within *sdh* genes confer selection advantages upon fluopyram application^17^.

Our study discloses variations in sensitivity of nematode genera towards fluopyram and provides evidence that this can be explained by a naturally occurring single amino acid difference in *Heterodera* spp. SDHC compared to other nematodes. Molecular understanding can moreover guide fluopyram use recommendations and the development of nematode management strategies in agricultural practices.

## Methods

### Fluopyram

Analytical grade fluopyram (Bayer AG, Monheim, Germany) was dissolved in dimethyl sulfoxide (DMSO) to obtain a stock solution and diluted to the appropriate working concentrations with water.

### Propagation and collection of nematodes

#### Caenorhabditis elegans

*Caenorhabditis* *elegans* N2 wild type (originally obtained from Prof. Einhard Schierenberg, University of Cologne Germany) and mutant RP2699 (kindly provided by Prof. Peter Roy, Ph.D., University of Toronto, USA) were maintained according to standard methods released by the *Caenorhabditis* Genetics Center, University of Minnesota, Minneapolis, MN 55,455 USA^18^.

#### Heterodera schachtii

*Heterodera* *schachtii* was multiplied and harvested in vitro on mustard (*Sinapsis alba* cv. Albatros) roots growing on Knop medium supplemented with 2% sucrose^19^. Cysts were collected in a Baermann funnel^20^ and hatching of larvae was stimulated by soaking cysts in 3 mM ZnCl_2_. The J2 were washed five times with water and the number of J2/10 µl was adapted to the respective experiment.

#### *Meloidogyne incognita* and *M. javanica*

Pepper seeds var. Feher (for *M.* *incognita*) and tomato seeds var. Rentita (for *M.* *javanica*) (Quedlingburger Saatgut GmbH, Aschersleben, Germany) were planted into 1 L plastic pots filled with 1,350 g of sandy loam soil (62.6% sand, 13% clay, 24.5% silt, 2.2% humus, pH 6.8). After emergence pepper or tomato plants were infested with a mixed population of 10,000 fully developed eggs and second stage juveniles (J2) of either *M.* *incognita* or *M.* *javanica.* Plants were kept for ten weeks in the greenhouse at 25 °C, 60–75% relative humidity, 60–80% field capacity and 14 h illumination under sodium vapor pressure lamps. The infested plants were then harvested and roots were washed-free of soil. Clean roots were incubated in an aeriated water bath to promote hatching of viable J2. Collection of J2 was carried out by filtration through multiple sieves (250 µm, 100 µm, 25 µm).

### PPN viability and recovery assay

#### Heterodera schachtii

To determine the sensitivity of *H.* *schachtii* to fluopyram, the nematodes were incubated for 48 h in a DMSO/water solution containing 0–50 ppm fluopyram. Control treatments received the same amount of DMSO as the 50 ppm variant. About 30 J2 were added to each well of a 96-well plate and incubated with the above described fluopyram concentrations or DMSO control for 48 h with gentle shaking of 30 rpm. For evaluation, 2.5% (v/v) final concentration of 1 M NaOH was added to each well and analyzed with a Leica DM4000 microscope equipped with an Olympus C-5050 digital camera. J2 remaining immobile upon NaOH stimulus were considered to be dead. In a parallel experiment, 10,000 J2 for each concentration were incubated for 48 h on a shaker applying a gentle speed of 30 rpm. After incubation, nematodes were washed in an 11 µm sieve with 500 ml sterile water and transferred to 96-well plates with flat bottom. J2 were then incubated for 6 days in water followed by evaluation with NaOH as described to obtain the percentage of recovered J2.

#### *Meloidogyne incognita* and *M. javanica*

J2 recovery assays were performed in vitro at room temperature. Fluopyram in DMSO was diluted with tap water to obtain 20 ml of the final test concentrations in 0.1% DMSO. A minimum of 10,000 J2 per treatment were added to each of the chemical solutions and incubated for 48 h on a plate shaker applying a gentle speed of 30 rpm. 48 h after incubation, J2 were transferred to a Büchner funnel to remove the chemical solution by vacuum filtration through 0.45 µm cellulose acetate filter disks. To ensure a complete removal of fluopyram, a volume of 250 ml tap water was added to each funnel and used to wash treated J2 by another step of vacuum filtration. Clean J2 were collected in fresh tap water. Two aliquots per treatment were transferred to 96-well plates and the number of mobile and immobile J2 was counted under a microscope. The activity of tested substances was calculated by comparing the number of immobilized J2 per treatment with that in the DMSO control. J2 were then incubated for additional 6 days followed by another assessment to record the percentage of recovered J2.

### Infection assays

#### *Meloidogyne incognita*/*M. javanica*

The virulence of treated J2 was tested in 6-well plates. Each well was filled with 5 ml dry silica sand, 30 lettuce seeds var. Attractie (SPERLI GmbH, Everswinkel, Germany), 2.5 ml tap water and approximately 300 J2. Plates were kept in the greenhouse at growing conditions described above and irrigation was carried out daily. J2 virulence was evaluated after two weeks by counting the number of root galls per well.

#### Heterodera schachtii

Infectivity and development of *H.* *schachtii* J2 was determined on *A.* *thaliana* Col-0. For the first assay, nematodes were incubated for 48 h in a DMSO/water solution containing up to 50 ppm fluopyram. After removing the chemical, J2 were incubated in water for further 6 days. After incubation, plants were inoculated with 50–60 J2. For the second assay, *A.* *thaliana* was grown on fluopyram- (0.06 ppm (equal to 0.15 µM) or DMSO-containing medium for 12 days. Plants were inoculated with 50–60 untreated J2 and kept in a growth chamber at 25 °C with a 16 h light and 8 h dark cycle. For both assay infection was determined by counting the number of adult males and females 14 days after inoculation.

### Adenylate energy charge (AEC value) assay

#### Sample preparation

Between 15,000 (*H.* *schachtii*) and 60,000 nematodes (*M.* *incognita* or *C.* *elegans*) of a mixed population were collected in 20 ml water (*H.* *schachtii* and *M.* *incognita*) or S-medium (*C.* *elegans*). Then, 20 ppm fluopyram or DMSO were added and nematodes were incubated at 21 °C on a rocking shaker applying a gentle movement. 48 h after incubation, nematodes were transferred to a vacuum filter/storage bottle system (430,767, Corning) to remove the chemical solution by vacuum filtration through 0.22 µm cellulose acetate filter disks. Then, nematodes were collected in 1 ml water and subsequently centrifuged at 12,300 rpm, 4 °C for 10 min. While the supernatant was discarded, the pellet was dissolved in 5% trichloracetic acid (TCA) and a 3 mm tungsten carbide ball (Qiagen, Cat. No. 69997) was added to the solution. Probes were inserted into a beat mill (MM300, Retsch, Haan, Germany) and shaken at 28 Hz for 1 min. Then, samples were incubated for 2 min on ice, before the shaking step was repeated (28 Hz, 1 min).

Afterwards, the tungsten carbide ball was removed, and each probe was sonified for 30 s on ice. Then, 150 µL HPLC buffer was added, which contained: 5 mM tetrabutylammonium hydrogen sulfate, 110 mM NaH_2_PO_4_, 40 mM Na_2_HPO_4_, 6.6% MeOH (pH was adjusted with NaOH to 6.5). Samples were centrifuged at 13,200 rpm, 4 °C for 10 min, afterwards the supernatant was filtered through a 0.45 µm filter and filled into HPLC vials.

#### High performance liquid chromatography

All samples were analyzed using an Agilent 1100 binary HPLC-System (Agilent, Waldbronn, Germany) equipped with online degasser (Agilent), column oven (Agilent), CTC HTS PAL autosampler (Chromtech, Idstein, Germany), and YMC-Pack Pro C18 50 × 4.0 mm i.d., 3 μm particle size column (YMC, Kyoto, Japan). The mobile phase consisted of 5 mM tetrabutylammonium hydrogen sulfate, 110 mM NaH_2_PO_4_, 40 mM Na_2_HPO_4_, 6.6% MeOH (pH was adjusted with NaOH to 6.5), with a flow rate of 1 ml min^−1^. As reference, a mixture of 10^−4^ M AMP, 10^−4^ M ADP, and 10^−4^ M ATP was used (approximate retention times were 2 min, 3.5 min and 6.2 min, respectively).

#### Calculation of the AEC value

After adenylate concentrations were quantitatively determined via an HPLC analysis, the AEC value was calculated with the following formula:

$$ AEC = \frac{ATP + 0.5 \, ADP}{{ATP + ADP + AMP}} $$

### Measuring succinate:ubiquinone reductase (complex II) activity

Succinate ubiquinone reductase activity was measured according to the method previously described by Barrientos et al. (2002)^21^.

Mitochondrial proteins were isolated from either housefly flight muscle, rat heart, earthworm or *C.* *elegans* by standard procedure using differential centrifugation^22^.

In brief, the assay was monitored at 600 nm using a Tecan M1000 Spectrophotometer (Tecan Group, Männedorf, Switzerland) in 384-well plates (Greiner Bio One, Kremsmünster, Austria) using 1 µg of mitochondrial protein per well in 50 mM KH_2_PO_4_ (pH 7.2), 4% DMSO (f.c.), 100 µM 2,6-dichlorophenolindophenol (DCPIP), 20 µM decylubiquinone, 1 µM antimycin and 0.33% f.c. butanedioic acid (succinate substrate). Antimycin was added to prevent electron transfer to complex III of the respiratory chain. Enzyme activity was measured in the presence of different inhibitor concentrations for 20 min and pIC_50_ values were calculated as negative logarithmic values of the half maximal inhibitory concentration (IC_50_).

### Sequence comparison

*Caenorhabditis* *elegans* sequences of the SDH subunits were retrieved from the UniProt database (uniprot.org)^23^ and have the following UniProt entries: Q09545/SDHB_CAEEL (SDHB), P41956/C560_CAEEL (SDHC), o62215/DHSD_CAEEL (SDHD). These protein sequences were used as queries to search for similar sequences in different nematode species as well as *Homo sapiens*, *Mus musculus* and *Rattus norvegicus* utilizing the basic local alignment search tool (BLAST) provided by the National Center for Biotechnology Information (NCBI; blast.ncbi.nlm.nih.gov)^24^ and WormBase (wormbase.org)^25^. The respective sequences of *H. schachtii* were extracted from in house genomic data^26^.

### *Caenorhabditis elegans* assays

#### Preparing OP50

*Escherichia coli* OP50 was incubated overnight in LB medium with shaking (150—200 rpm) at 37 °C. Cultured OP50 in LB medium was transferred into a 50 ml tube. The tube was centrifuged at 4,000 rpm for 5 min, and the supernatant was removed. OP50 pellets were stored at − 80 °C until use.

#### Synchronization of *C. elegans*

Nematodes cultured on NGM agar plates [3 g NaCl, 17 g agar, 2.5 g peptone, 1 ml 5 mg/ml cholesterol in EtOH, 1 ml 1 M KPO_4_ buffer pH 6.0 [108.3 g KH_2_PO_4_, 35.6 g K_2_HPO_4_, H_2_O to 1 L], 1 ml 1 M MgSO_4_, 1 ml 1 M CaCl_2_, H_2_O to 1 L] were collected in M9 buffer [3 g KH_2_PO_4_, 6 g Na_2_HPO_4_, 5 g NaCl, 1 ml 1 M MgSO_4_, H_2_O to 1 L] and transferred into a 50 ml tube. The tube was put on ice to pellet the nematodes and the supernatant was removed. 4 ml bleach solution [1 ml 6% NaOCl, 4 ml 1 M NaOH, 5 ml ddH_2_O] was added to the pellet. After 10 min gentle shaking, the nematode cuticles were degraded and eggs were released. The tube was refilled to 50 ml with M9 buffer and centrifuged for 3 min at 1,300 rpm. After removing the supernatant, the tube was refilled to 35 ml with M9 buffer. The eggs were washed three times with M9 buffer by the same procedure. The washed eggs were suspended in 15 ml M9 buffer and incubated overnight on a nutator at 22 °C. The supernatant was discarded after centrifugation at 2,000 rpm for 1 min to remove dauer pheromones.

#### Inhibition and recovery assay

Approx. 40 synchronized L1s in 5 µl NGM were added to each well of a 96-well plate. Frozen *E. coli* OP50 pellet was diluted to OD_600nm_ = 7.8 with NGM and 15 µl OP50 solution was added to each well. 100 µl of each prepared fluopyram solution was added to each well to obtain final concentrations of 10, 20 and 50 ppm. The highest final concentration of DMSO was 0.25% (v/v). The plate was incubated for 48 h at 22 °C without shaking and subsequently the number of mobile and immobile nematodes were counted under the microscope. Nematodes were washed three times with 200 µl NGM. After that the supernatant was removed expect approximately 20 µl. Frozen *E. coli* OP50 pellet was diluted to an OD_600nm_ of 1.14 with NGM and 100 µl OP50 solution was added to each well. The plate was incubated for another 48 h at 22 °C without shaking. The number of mobile/immobile nematodes were counted under the microscope to determine the number of nematodes that recovered from the fluopyram treatment.

#### Development assay

Approximately 40 synchronized L1s in 20 µl NGM were added to each well of a 96-well plate. Frozen *E. coli* OP50 pellet was diluted to an OD_600nm_ of 1.4 with NGM and 88 µl OP50 solution was added to each well. 12 µl of each prepared fluopyram solution was added to each well to obtain final concentrations of 0.1, 1 and 10 ppm. The highest final concentration of DMSO was 0.05% (v/v). The number of matured nematodes from synchronized L1s was counted under the microscope after 144 h incubation.

### Molecular modeling of nematode SQR sequences

All molecular modeling studies were carried out within the Maestro software suite (Schrödinger Release 2019-1, Maestro, Schrödinger, LLC, New York, 2019). Construction and refinement of the nematode SDH models was realized by Maestro’s advanced homology modeling tool. From a series of complex II co-crystal structures of the parasitic worm *Ascaris suum* the PDB ID 4YSY was chosen because of the high chemical similarity of the co-crystallized inhibitor with fluopyram^27,28^. Fluopyram was fitted onto the respective positions of the amide group and the o-trifluoromethyl-phenyl moiety, while the chlorpyridyl was placed in a hydrophobic pocket formed by a nematode-specific tryptophan (sidechain).

### Statistics

Results are displayed as means + /− standard error (SE). At least three biological replicates of each experiment were performed including the number of technical replicates indicated in the results section. Significant differences were determined using one-way analysis of variance (ANOVA) and an appropriate post-hoc test (*p* < 0.05) (SigmaPlot 12.5, Systat Software, Inc., USA).

### Ethics declaration

For the experiments involving plants and vertebrates, all local, national or international guidelines and legislation were adhered to in the study.

## Supplementary Information

Supplementary Information.

## Data Availability

All data generated or analyzed during this study are included in this published article and the supplementary material. More details are available from the corresponding authors on reasonable request.
