# Author Correction: Mode of action of fluopyram in plant-parasitic nematodes

**DOI:** 10.1038/s41598-023-40484-z

**Published:** 2023-08-23

**Authors:** A. Sylvia S. Schleker, Marc Rist, Christiane Matera, Arunas Damijonaitis, Ursel Collienne, Koichi Matsuoka, Samer S. Habash, Katja Twelker, Oliver Gutbrod, Corinna Saalwächter, Maren Windau, Svend Matthiesen, Tatyana Stefanovska, Melanie Scharwey, Michael T. Marx, Sven Geibel, Florian M. W. Grundler

**Affiliations:** 1 Molecular Phytomedicine, University of Bonn, Karlrobert‑Kreiten‑Straße 13, 53115 Bonn, Germany; 2 Research and Development, CropScience Division, Bayer AG, Alfred‑Nobel‑Str. 50, 40789 Monheim am Rhein, Germany; 3 Department of Entomology, National University of Life and Environmental Sciences, Kyiv, 03041 Ukraine; 4 Present Address: BASF Vegetable Seeds, Napoleonsweg 152, 6083 AB Nunhem, The Netherlands

Correction to: *Scientific Reports* 10.1038/s41598-022-15782-7, published online 13 July 2022

The original version of this Article contained an error in the Competing Interests statement.

“The authors declare no competing interests.”

now reads:

“This study was a research cooperation between the University of Bonn and Bayer AG. Bayer AG sponsored parts of the study. M.R., A.D., U.C., K.T., O.G., C.S., M.W., S.M., M.S., M.T.M., and S.G. were employees of Bayer AG during this research and supported the manuscript as indicated in the contribution section. S.S.H. was employed by the University of Bonn during his contributions to this research and is now an employee of BASF Vegetable Seeds. Any statements, findings or conclusions made in this publication are those of the authors and are not influenced by the sponsor or employer.”

The original article has been corrected.

