# Supplementary material for: Mode of action of fluopyram in plant-parasitic nematodes
Source: Sci Rep. 2022 Jul 13;12:11954. doi: 10.1038/s41598-022-15782-7 (PMC9279378; doi:10.1038/s41598-022-15782-7)
Supplement: Supplementary file 1 — Supplementary Information. [file 41598_2022_15782_MOESM1_ESM.pdf]

Supplementary Material

Mode of Action of Fluopyram in Plant-Parasitic Nematodes

A. Sylvia S. Schleker[1,*], Marc Rist[2,*], Christiane Matera[1], Arunas Damijonaitis[2], Ursel Collienne[2], Koichi Matsuoka[1], Samer S. Habash[1,4], Katja Twelker[2], Oliver Gutbrod[2], Corinna Saalwächter[2], Maren Windau[2], Svend Matthiesen[2], Tatyana Stefanovska[3], Melanie Scharwey[2], Michael T. Marx[2], Sven Geibel[2] and Florian M. W. Grundler[1]

[1]Molecular Phytomedicine, University of Bonn, Karlrobert-Kreiten-Straße 13, 53115 Bonn, Germany

[2]Research and Development, CropScience Division, Bayer AG, Alfred-Nobel-Str.50, 40789 Monheim am Rhein, Germany

[3]Department of Entomology, National University of Life and Environmental Sciences, 03041 Kyiv, Ukraine

[4]Present address: BASF Vegetable Seeds, Napoleonsweg 152, 6083 AB Nunhem, The Netherlands

*Correspondence: sylvia.schleker@uni-bonn.de and marc.rist@bayer.com

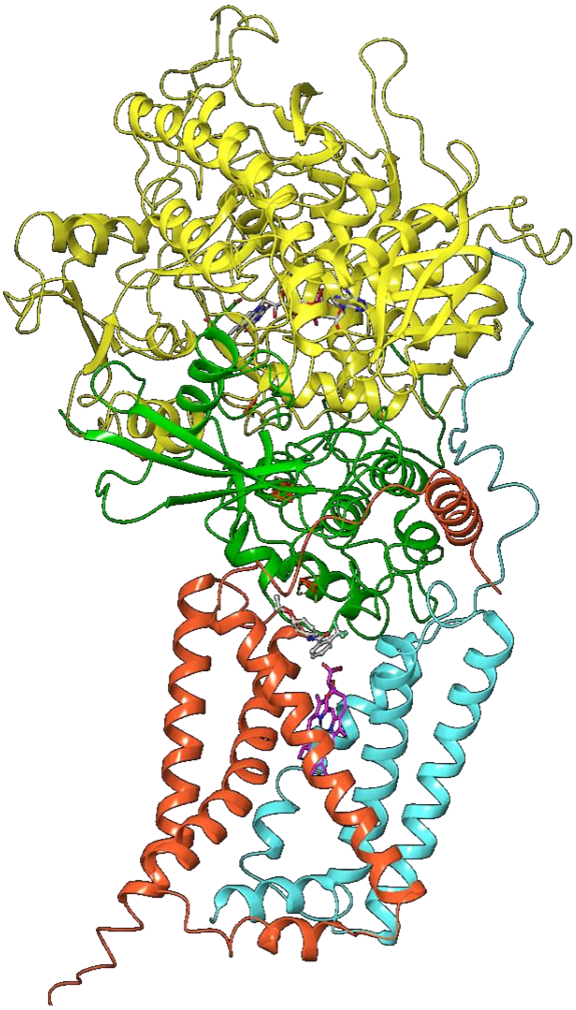

Figure S1. Overall fold of *Caenorhabditis elegans* SDH homology model displayed with heme moiety and manually docked fluopyram inhibitor in its catalytic site. Chain SDHA (yellow) carries the FAD co-substrate (partially occluded), SDHB (green) hosts two iron-sulphur clusters (orange/yellow), the SDHC (brown) and SDHD (cyan) units jointly bind the heme moiety (magenta) and the fluopyram inhibitor (grey).

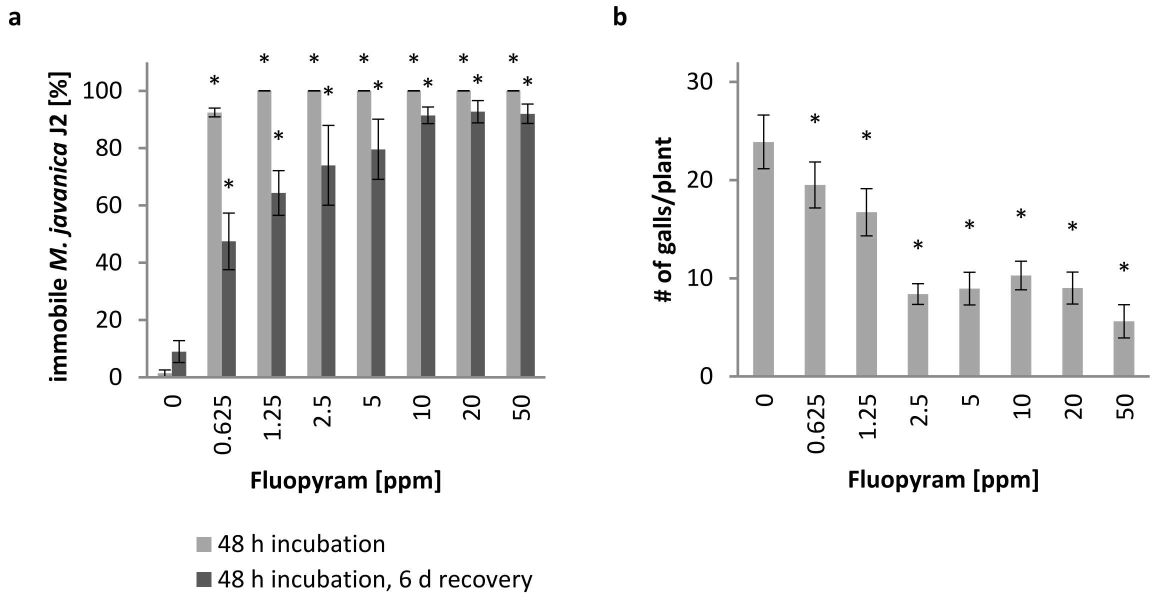

Figure S2: Fluopyram is lethal for *Meloidogyne javanica* (a) and pre-treatment of J2 with fluopyram reduces *M. javanica* caused gall formation (b). (a) *M. javanica* J2 were exposed to different concentrations of fluopyram or DMSO control for 48 h. Subsequently, nematodes were washed and incubated in water for further six days. The number of immobile nematodes were counted at these two time points. Values represented as mean ± SE of three independent biological replicates (n = 6). Asterisks indicate significant differences to control according to Student-Newman-Keuls method ($p<0.05$). **(b)** *M. javanica* J2 were incubated in different concentrations of fluopyram or DMSO as control for 48 h. Subsequently, the compound and DMSO were removed, the J2 were incubated in water for further six days and then used to inoculate lettuce. The number of galls was determined. Bars display mean ± SE of three independent biological replicates (n = 18). Asterisks indicate significant differences to control according to Student-Newman-Keuls method ($p<0.05$).
